# Investigating the synaptic trafficking of AD Biomarker Neuronal Pentraxin 2: Implications for Interneuron and Cholinergic Circuit Dysfunction in Alzheimer’s Disease

**DOI:** 10.1002/alz.095172

**Published:** 2025-01-09

**Authors:** Seung‐Eon Roh, Mira Swartzlander, Kathryn Cheong, Paul F Worley

**Affiliations:** ^1^ Johns Hopkins University School of Medicine, Baltimore, MD USA

## Abstract

**Background:**

Deficits in interneuron and cholinergic circuits are noted in AD pathology, yet the precise mechanisms of their contribution to cognitive decline in the disease remain elusive. Neuronal Pentraxin 2 (NPTX2), a sensitive marker for synaptic activity and AD progression, is an immediate early gene expressed by pyramidal neurons that functions at excitatory synapses on Parvalbumin interneurons (PV‐IN) to cluster AMPA receptors and strengthen circuit inhibition. NPTX2 is later shed from some synapses into the cerebrospinal fluid (CSF), where reduced NPTX2 levels inversely correlate with hippocampal volume and cognitive performance in individuals with AD/MCI. Recent studies employing NPTX2‐SEP (Super‐Ecliptic pHluorin) as a marker of its synaptic trafficking have suggested that the diurnal oscillation of NPTX2 trafficking is important for circuit refinement, which is crucial for ensemble formation and memory updating.

**Method:**

We aim to investigate the mechanisms of NPTX2 synaptic trafficking by utilizing two‐photon imaging of NPTX2‐SEP in the somatosensory cortex while chemo‐ and optogenetically manipulating PV‐IN and cholinergic circuits, specifically the basal forebrain (BF) and cortical intrinsic cholinergic neurons.

**Result:**

We found that reducing PV‐IN activity leads to rapid shedding of NPTX2, whereas increasing PV‐IN activity results in elevated accumulation of NPTX2 at excitatory synapses on PV‐IN. This suggests that PV‐IN activity is a critical determinant for NPTX2 synaptic exocytosis and shedding and supports a model akin to spike‐timing‐dependent plasticity, wherein synapses are strengthened with correlated pre‐ and postsynaptic activity and weakened when presynaptic activity is not associated with postsynaptic activity. Direct activation of BF cholinergic neurons had minimal effect on NPTX2‐SEP. However, consistent with the observation that activation of nicotinic and muscarinic ACh receptors (AChR) can respectively increase and decrease cortical PV‐IN activity, chemogenetic activation of BF cholinergic neurons with mAChR blocker induced NPTX2 shedding, while nicotine also moderately induced its shedding. Additionally, chemo‐ and optogenetic activation of cortical intrinsic cholinergic interneurons reduced PV‐IN activity and induced NPTX2 shedding.

**Conclusion:**

These results underscore the significant roles of the ACh‐PV pathway in regulating NPTX2 synaptic trafficking and implicate deficits in NPTX2 shedding and circuit refinement due to the failure of the ACh‐PV pathway as a mechanism for cognitive decline in AD.

